# Supplementary material for: Benchmarking Eliminative Radiomic Feature Selection for Head and Neck Lymph Node Classification
Source: Cancers (Basel). 2022 Jan 18;14(3):477. doi: 10.3390/cancers14030477 (PMC8833684; doi:10.3390/cancers14030477)
Supplement: Supplementary file 1 [file cancers-14-00477-s001.zip › cancers-1513232-supplementary.pdf]

cancers

MDPI

Supplementary file

Supplementary Table S1. Summary of all 120 radiomic features extracted using the open-source PyRadiomics library [24,25].

**First Order Statistics**	**Shape-based (3D)**
Energy	Mesh Volume
Total Energy	Voxel Volume
Entropy	Surface Area
Minimum	Surface Area to Volume Ratio
10th percentile	Sphericity
90th percentile	Compactness 1
Maximum	Compactness 2
Mean	Spherical Disproportion
Median	Maximum 3D Diameter
Interquartile Range	Maximum 2D Diameter (Slice)
Range	Maximum 2D Diameter (Column)
Mean Absolute Deviation	Maximum 2D Diameter (Row)
Robust Mean Absolute Deviation	Major Axis Length
Root mean Squared	Minor Axis Length
Standard Deviation	Least Axis Length
Skewness	Elongation
Kurtosis	
Variance	
Uniformity	
**Gray Level Co-occurrence Matrix**	**Shape-based (2D)**
Autocorrelation	Mesh Surface
Joint Average	Pixel Surface
Cluster Prominence	Perimeter
Cluster Shade	Perimeter to Surface Ratio
Cluster Tendency	Sphericity
Contrast	Spherical Disproportion
Correlation	Maximum 2D Diameter
Difference Entropy	Major Axis Length
Difference Variance	Minor Axis Length
Joint Energy	Elongation
Joint Entropy	
Informational Measure of Correlation 1	
Informational Measure of Correlation 2	
Inverse Difference Moment	
Maximal Correlation Coefficient	
Inverse Difference Moment Normalized	
Inverse Difference	
Inverse Difference Normalized	
Inverse Variance	
Maximum Probability	
Sum Average	
Sum Entropy	
Sum of Squares	
Sum of Squares	
**Gray Level Run Length Matrix**	**Gray Level Size Zone Matrix**

Short Run Emphasis	Small Area Emphasis
Long Run Emphasis	Large Area Emphasis
Gray Level Non-Uniformity	Gray Level Non-Uniformity
Gray Level Non-Uniformity Normalized	Gray Level Non-Uniformity Normalized
Run Length Non-Uniformity	Size-Zone Non-Uniformity
Run Length Non-Uniformity Normalized	Size-Zone None-Uniformity Normalized
Run Percentage	Zone Percentage
Gray Level Variance	Gray Level Variance
Run Variance	Zone Variance
Run Entropy	Zone Entropy
Low Gray Level Run Emphasis	Low Gray Level Zone Emphasis
High Gray Level Run Emphasis	High Gray Level Zone Emphasis
Short Run Low Gray Level Emphasis	Small Area Low Gray Level Emphasis
Short Run High Gray Level Emphasis	Small Area High Gray Level Emphasis
Long Run Low Gray Level Emphasis	Large Area Low Gray Level Emphasis
Long Run High Gray Level Emphasis	Large Area High Gray Level Emphasis
**Gray Level Dependence Matrix**	**Neighboring Gray Tone difference Matrix**
Small Dependence Emphasis	Coarseness
Large Dependence Emphasis	Contrast
Gray Level Non-Uniformity	Busyness
Dependence Non-Uniformity	Complexity
Dependence Non-Uniformity Normalized	Strength
Gray Level Variance	
Dependence Variance	
Dependence Entropy	
Low Gray Level Emphasis	
High Gray Level Emphasis	
Small Dependence Low Gray Level Emphasis	
Small Dependence High Gray Level Emphasis	
Larger Dependence Low Gray Level Emphasis	
Large Dependence High Gray Level Emphasis	
